# The Height-Width-Depth Ratios of the Intervertebral Discs and Vertebral Bodies in Adolescent Idiopathic Scoliosis vs Controls in a Chinese Population

**DOI:** 10.1038/srep46448

**Published:** 2017-04-18

**Authors:** Hao Chen, Tom P. C. Schlösser, Rob C. Brink, Dino Colo, Marijn van Stralen, Lin Shi, Winnie C. W. Chu, Pheng-Ann Heng, René M. Castelein, Jack C. Y. Cheng

**Affiliations:** 1 Department of Computer Science and Engineering, The Chinese University of Hong Kong, Shatin, Hong Kong; 2 Department of Orthopedic Surgery, University Medical Center, Utrecht, The Netherlands; 3 Image Sciences Institute, University Medical Center, Utrecht, The Netherlands; 4 Department of Medicine and Therapeutics, The Chinese University of Hong Kong, Shatin, Hong Kong; 5 Department of Imaging and Interventional Radiology, Prince of Wales Hospital, The Chinese University of Hong Kong, Shatin, Hong Kong; 6 Department of Orthopaedics and Traumatology, Prince of Wales Hospital, The Chinese University of Hong Kong, Shatin, Hong Kong

## Abstract

Adolescent idiopathic scoliosis (AIS) patients have been reported to be taller and more slender than normal controls, suggesting less mechanical stiffness of their trunk and spine. For assessment of mechanical stiffness, to our best knowledge this is the first to study height-width-depth relations at the level of the individual vertebra and disc in 3-D and to evaluate its relation with the Cobb angle severity. A unique series of high-resolution pre-operative computed tomographic (CT) scans of a total of 105 Chinese patients with moderate to severe AIS and 11 age-matched non-scoliotic controls were used for this study. It was found that some geometric relations differed between primary thoracic curves, secondary curves and normal controls at the individual affected vertebra and disc level. The scoliotic discs in the primary curves were relatively more slender (taller and thinner) than in secondary curves and as compared to controls. In the lumbar spinal area, the vertebral bodies were more slender in the primary as well as secondary AIS curves as compared to the controls. Therefore, if all material properties remain the same, our finding indicates that scoliotic spines may be mechanically less stiff than normal spines. No significant correlation between any of the measures and Cobb angle severity was found.

A number of clinical and radiological studies have reported that adolescent idiopathic scoliotic (AIS) patients have different body dimensions than normal matched controls^1^,^2^,^3^,^4^,^5^,^6^,^7^. They were found to be relatively taller and have a lower body mass index (BMI), suggesting a less stable configuration of their spine. Different anthropometric parameters, such as a lower weight, a lower BMI and longer arm span, were found correlated with a larger curve Cobb angle magnitude^6^,^7^. A large population study found that below normal BMI is associated with severity of spinal deformities, whereas above-normal BMI apparently has a protective effect^8^. It is understandable that external body dimension does not necessarily match with the internal anatomical configuration^9^,^10^. Conversely, some studies suggest a higher BMI at presentation to a scoliosis clinic is associated with larger Cobb angles^11^,^12^,^13^.

In 1984, Schultz *et al*. introduced a method to study height-width-depth relationships of the T5-L3 spinal region in asymptomatic adolescents using conventional radiographs^14^. They observed that the spines of girls were significantly more slender (i.e., taller and/or thinner shape) than those of boys. Conventional studies, however, are constrained by the two-dimensional radiographs and are unsuitable for studying the true 3-D anatomy of the scoliotic spine^14^,^15^,^16^,^17^,^18^. Modern 3-D imaging techniques provide the opportunity to assess spine morphology in full detail at the vertebra as well as the disc level. To the best of our knowledge, no studies have looked at height-width-depth relationships at the individual vertebra and disc level between patients with idiopathic scoliosis and normal controls. The objective of this study was to analyze the spinal dimensions of the AIS spine in 3-D *in vivo* by measurement of the total length of the spine, and height-width-depth ratios of the vertebral bodies and intervertebral discs at individual vertebral level by using a series of high resolution computed tomography (CT) scans obtained pre-operatively for navigation surgical guidance purposes. These findings were compared with a non-scoliotic control group of which CT scans were obtained for indications not related to their spines.

## Results

### Demographics of study population

**Figure 1:**
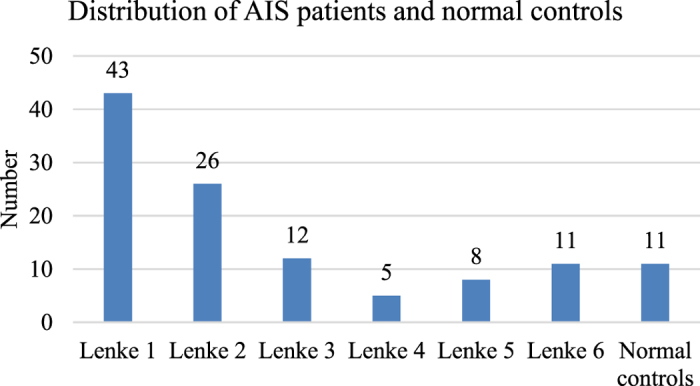
Distribution of normal controls and AIS patients enrolled in this study.

Demographics of subjects in our experiments are shown in Table 1 and Fig. 1. The mean age of the 105 AIS patients at diagnosis was 16.2 ± 2.7 years. Mean Cobb angle of the main thoracic and thoracolumbar curves was 66.9 ± 15.0° and 44.0 ± 16.1°, respectively. The control cohort consisted of 11 race-sex and age matched children who had undergone full-body CT for trauma screening, and they had no abnormalities of their spine or pelvis. All patients and controls were from Chinese population.

**Figure 2:**
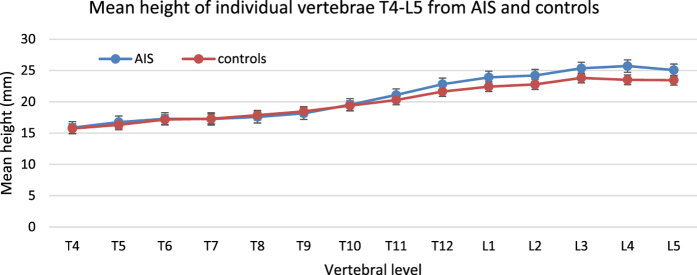
Mean height of individual vertebrae T4-L5. The error bar indicates the standard error.

**Figure 3:**
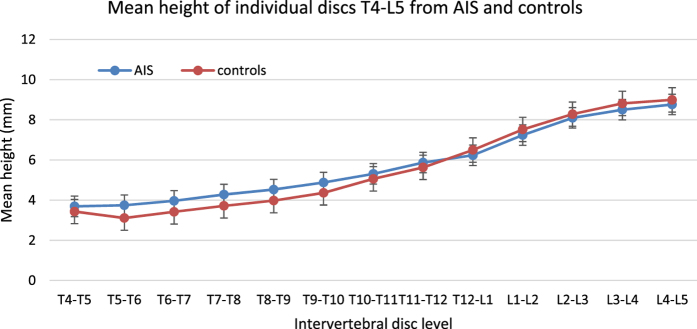
Mean height of individual discs T4-L5. The error bar indicates the standard error.

3-D morphological parameters including height, width, and depth were analyzed on a total of 1470 vertebrae and 1365 discs of AIS patients and 154 vertebrae and 143 discs of normal controls. The total spinal length, defined as the summation of superior-inferior central heights including vertebrae and discs from T4 to L5, of scoliosis and normal spine were 365.6 ± 20.1 mm and 352.8 ± 31.7 mm, respectively (*p* = 0.12). The mean heights of individual vertebra and disc from T4 to L5 are shown in Figs 2 and 3, respectively.

### Relative dimensions of the spine in AIS patients and controls

In the thoracic spine, significant differences in vertebral and disc dimensions were observed between the subgroups. In more detail, there were no significant differences in vertebral body dimensions between primary thoracic AIS curves (Lenke 1–4) and controls (Table 2). The intervertebral discs in the thoracic spine, however, showed significantly greater height-depth ratio as well as trend towards greater height-width and width-depth ratio. The vertebrae were relatively more slender in the secondary thoracic curves (Lenke 5–6) as compared to the vertebrae in the primary thoracic curves (Lenke 1–4), whereas the discs were relatively more slender in the primary thoracic curves as compared to the secondary thoracic curves. In our study, the slenderness is defined as a relatively taller and/or thinner shape.

In the lumbar spinal area, vertebral body dimensions (*R*_*hw*_ and *R*_*hd*_) differed significantly between the groups (Table 3), whereas no differences in disc dimensions were observed. Further analyses showed that *R*_*hw*_ and *R*_*hd*_ were significantly greater in the primary as well as secondary AIS curves as compared to the controls.

Correlations between Cobb angle and relative spinal dimensions of vertebrae and discs in different sections of AIS subgroups were analyzed. No significant, relevant correlations (*p* < 0.05 and *r* > 0.3) were found between the ratios and Cobb angle.

## Discussion

Idiopathic scoliosis patients have been previously reported to be taller and more slender than their peers^1^,^2^,^3^,^4^,^5^,^6^,^7^. The premise underlying these studies is that this difference in body habitus implies less mechanical stability of the trunk and spine, thus predisposing to the development or progression of spinal deformity. External traits, however, do not necessarily correlate with internal characteristics. For spinal stability, the dimensions of the vertebral column itself seem more relevant to predict its mechanical stability characteristics. This study is, to the best of our knowledge, the first to study spinal dimensions at the level of the vertebral body and disc, and compare between patients with idiopathic scoliosis and a small group of matched normal controls. For this purpose a series of high resolution CT scans of scoliotic patients, obtained for navigation purposes, were used and compared with an age-and-sex matched cohort without scoliosis, obtained for trauma screening. Patients with idiopathic scoliosis appeared to have different spinal morphologies than non-scoliotic controls. From our data follows that in primary thoracic idiopathic scoliosis, the vertebrae have similar height-width, height-depth as well as width-depth ratio’s, whereas the discs are more slender, and therefore more susceptible to biomechanical impact, as compared to asymptomatic adolescents.

Before this study, it was already known that under certain circumstances (when there is imbalance between the acting force and its compensating mechanism), an excess of forces that act on the spine could lead to the development of spinal deformity: axial compression of the adolescent spine might lead to a deformity known as Scheuermann’s kyphosis^19^ and similarly, excess of anteriorly directed shear loads could result in spondylolisthesis^20^,^21^. Posterior shear loads were shown to play a role in the rotational stiffness of the human spine due to its unique biomechanical loading^22^,^23^,^24^,^25^,^26^. It is hypothesized that the development of AIS could result from disturbance of the delicate balance between stability of the fully upright human spine and stability challenging deforming forces. In this study, spinal dimensions and the projected stability are evaluated in adolescent idiopathic scoliosis patients and compared to normal matched controls.

This is not the first study that focusses on spinal dimensions. In the last decades, differences in body height between AIS patients and normal controls were confirmed in multiple studies^1^,^2^,^3^. Significant height increase was found in the head, trunk and lower extremities within AIS patients as compared to controls^3^. Moreover, on conventional 2-D radiographs, children with AIS were taller than their non-scoliotic peers of the same chronological age^15^,^27^. Based on two-dimensional imaging, the literature suggests that spinal length of children affected by AIS is greater than that of age-matched non-scoliotic children^16^,^17^,^18^. It is difficult to assess the complex three dimensional changes that take place in scoliosis on two dimensional X-Rays. These changes include significant alterations of the sagittal plane that obviously influence height measurements on the anterior *versus* posterior side of the vertebral bodies and intervertebral discs^28^. Our study takes these changes into account by measurement of height at the level of the center of mass of individual endplates in the corrected transverse plane. It demonstrates that the spine in primary thoracic idiopathic scoliosis may be less mechanically stable than the normal spine, at the level of the dimensions of the intervertebral disc.

The CT images of AIS patients were taken in prone position pre-operatively, for navigation purposes. CT images of the controls were acquired for indications other than the spine in a supine position. For this reason, positioning may have influenced our results as it likely affects the 3-D morphology of the discs. Sub-analyses on the individual bony vertebra, which are not position dependent, however, also show significant differences in height-width-depth dimensions between primary and secondary curves and between secondary curves and controls. However, no significant differences in bony height-width-depth dimensions were found between the primary thoracic curves and controls.

In conclusion, the comparison analysis on the height-width-depth ratios between AIS patients and normal controls showed that the thoracic spine in primary thoracic idiopathic scoliosis tends to be more slender than the thoracic spine of non-scoliotic controls, as a result of different disc dimensions. This could be related to a natural variation which predisposes these individuals to develop a thoracic spinal deformity, or it could imply the existence of abnormal growth and bone metabolism as a result of imbalances in the biomechanical forces acting on the spine during the peri-pubertal growth spurt that may contribute to the etio-pathogenesis of AIS.

## Methods

### Study population

The research protocol was approved by the institutional clinical research ethics committee of Prince of Wales Hospital, Shatin, Hong Kong and conducted in compliance with Declaration of Helsinki. All subjects enrolled in this study provided written informed consent.

High resolution CT scans were acquired from April 2010 to December 2016 for intraoperative navigation surgery for posterior pedicle screw based instrumentation in patients with moderate to severe AIS. CT scans of the normal controls were from patients investigated for other medical conditions without any spinal deformity. All incomplete scans (not showing at least T4 to L5) were excluded and race, sex and age-matching was performed between AIS subjects and normal controls. A total of 105 AIS patients scheduled for surgery and 11 age-matched controls were enrolled in this study with institutional IRB approvals. Scans of AIS patients were obtained in prone position, mimicking the position in surgery, with the arms on the side (slice thickness 0.625 mm, in-plane resolution 0.352 mm/pixel, 64-slice multi-detector CT scanner, GE Healthcare, Chalfont, St. Giles, United Kingdom), whereas scans of controls were obtained in the supine position with the same specifications. Conventional radiographs including standing posterior-anterior, lateral and supine bending radiographs, were taken at the same time as CT scans^18^,^29^,^30^. Standard X-Rays of scoliotic cases were reviewed by one experienced investigator and the curve type was classified according to the Lenke classification plus recording of Cobb angles of the major curves^31^,^32^.

**Figure 4:**
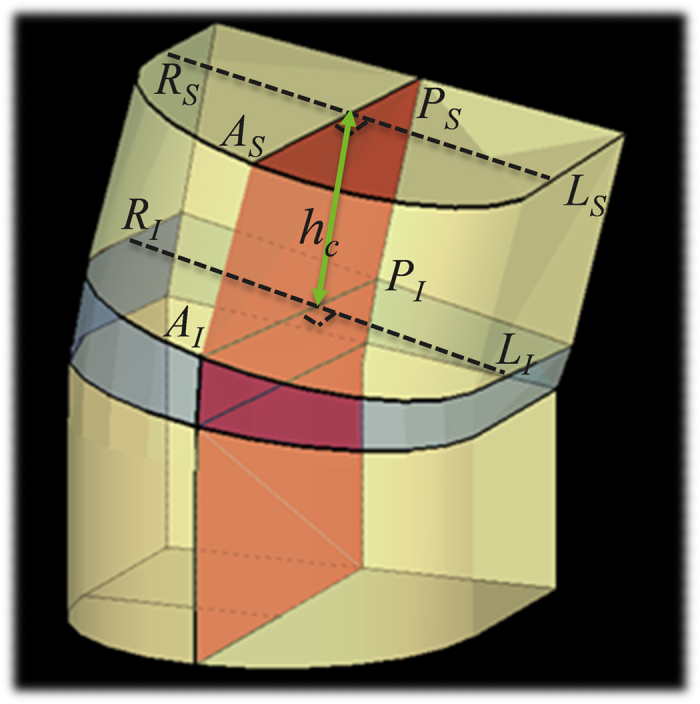
Illustration of spinal dimensions (take the vertebra for example). *A, P, R, L, S, I* indicate *anterior, posterior, right, left, superior* and *inferior* respectively.

Spinal dimensions were measured on the acquired CT scans for different spinal areas using the semi-automatic analysis method as described below. Relative spinal dimensions of vertebrae and discs as defined in Fig. 4 (we take the vertebra for example, same as the disc) were compared between primary thoracic AIS curves (Lenke 1–4) *versus* secondary thoracic AIS curves (Lenke 5–6) *versus* controls.

### Semi-automatic analysis method

**Figure 5:**
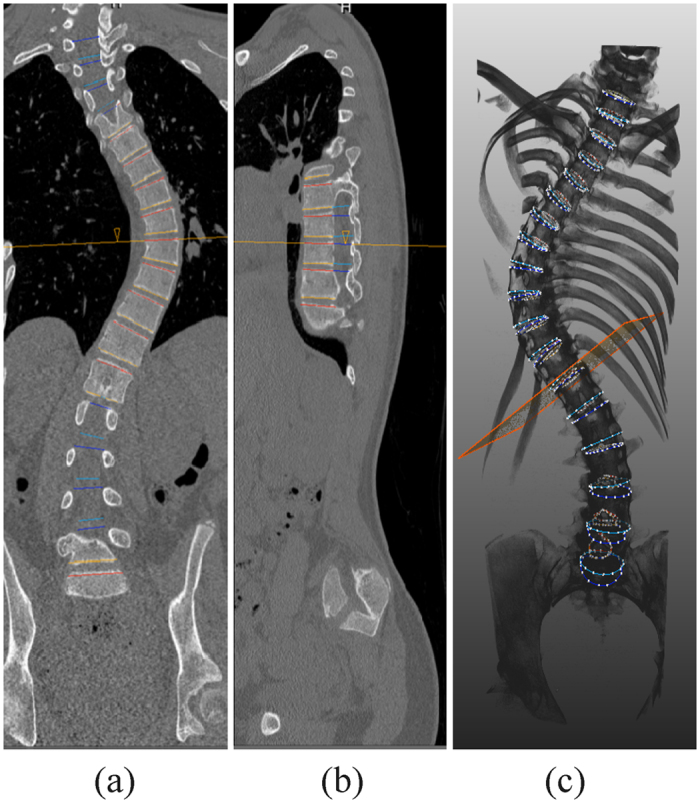
Example of semi-automatic analysis of the vertebrae and intervertebral discs on high resolution CT scans. (**a**,**b**) Represent the delineation of the upper/lower endplates in sagittal and coronal planes, respectively. (**c**) An example of a complete 3-D reconstruction and coordinate system based on the delineation and segmentation.

By application of a previously validated semi-automatic image processing technique, complete 3-D reconstructions of all vertebrae and intervertebral discs from T4 to L5 from CT scans were acquired using 3-D endplate vectors (Fig. 5)^28^. Due to incomplete visualization in the upper thoracic curve of several subjects, vertebrae and discs above T4 were not included in the final analysis. The endplate vectors were used as reference for determining the true anterior, posterior and lateral aspects per endplate of each vertebra and disc, taking inter-vertebral torsion, inter-vertebral rotation as well as sagittal and coronal tilting of each individual endplate into account. Finally, height, width, depth and corresponding ratios of each individual vertebra and disc were calculated as well as the total spinal length:

#### Height

Superior-inferior height was defined by *h*: the central height from the center of mass in the upper endplate to the center mass in the lower endplate; *S* and *I* stand for superior and inferior, *COM*_*s*_ and *COM*_*I*_ represent the center of mass in the upper (superior) and the lower (inferior) endplate, respectively and 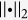 denotes the Euclidean distance.


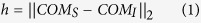


#### Width

The mean value between the left-right width in the upper and lower endplate. *L* and *R* stand for left and right, respectively.


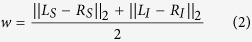


#### Depth

The mean value between the anterior-posterior axial depth in the upper and lower endplate. *A* and *P* stand for anterior and posterior, respectively.


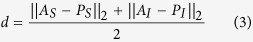


#### Length ratio parameters

The following ratios were calculated in the vertebrae and discs, separately. For practical purposes, slenderness was defined as greater *R*_*hw*_ or *R*_*hd*_ (taller or thinner).


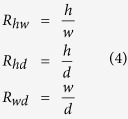


#### Total Spine Length T4-T5

The summation of the central height of all vertebrae and discs in the entire spine from T4 to L5. Where *L* denotes the total curve length, *L*_*v*_ and *L*_*i*_ denote the total curve length of vertebrae and intervertebral discs, respectively, *h*_*v*_ is the central height of vertebrae *v, h*_*i*_ is the central height of disc *i, N*_*v*_ and *N*_*i*_ are the total number of vertebrae and intervertebral discs, respectively.


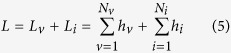


As verified in a previous study^28^, the mean error of length measurements on vertebral bodies and discs was 0.2 ± 0.1 mm. Intraclass Correlation Coefficients (ICC) for intra- and inter-observer reliabilities were 0.99 (95% confidence interval: 0.99–1.00) and 0.99 (0.98–1.00) for length measurement, respectively.

### Statistical analysis

SPSS 22.0 (SPSS Inc, Chicago, IL) was used for statistical analyses. Mean and standard deviation (SD) were calculated. Potential outliers were searched and there were no outliers identified in our study. Based on the mean measurement error of 0.2 mm, precision of height-width-depth ratios can be assumed to be more accurate than 1 decimal (for example, 20.6 mm height, 30.6 mm width, *R*_*hw*_ = 0.67). Therefore, to balance precision and simplicity, we kept 2 decimal places for ratios. Independent sample t-test was utilized for analysis between AIS patients and normal controls, because the two groups of subjects were independent from one another. Analysis of variance (ANOVA) was used to test for differences between the 3 groups including Lenke 1–4 *versus* Lenke 5–6 *versus* controls. Kolmogorov-Smirnov tests were used to test for normality of data distribution and confirmed that the data was normally distributed. For the group of AIS patients, ratios were analyzed in relation to the Cobb angles. Pearson’s correlation coefficient *r* was used for correlation relationship analysis. *p*-value < 0.05 was considered as statistically significant.

## Additional Information

**How to cite this article:** Chen, H. *et al*. The Height-Width-Depth Ratios of the Intervertebral Discs and Vertebral Bodies in Adolescent Idiopathic Scoliosis vs Controls in a Chinese Population. *Sci. Rep.* **7**, 46448; doi: 10.1038/srep46448 (2017).

**Publisher's note:** Springer Nature remains neutral with regard to jurisdictional claims in published maps and institutional affiliations.

## Figures and Tables

**Table 1:** Demographics of AIS patients and normal controls enrolled in this study (SD for standard deviation).

Demographics		AIS all n = 105	Primary thoracic n = 86	Secondary thoracic n = 19	Normal controls n = 11
Age (years)	Range	10–26	10–26	13–20	11–17
	Mean ± SD	16.2 ± 2.7	16.2 ± 2.9	16.1 ± 1.9	14.1 ± 2.3
Girls, n (%)		89 (85%)	73 (85%)	16 (84%)	8 (73%)
Main Thoracic curve Cobb angle	Range (°)	23–110	46–109	23–110	N/A
	Mean ± SD (°)	66.9 ± 15.0	69.4 ± 12.3	55.9 ± 20.2	N/A
Thoracolumbar curve Cobb angle	Range (°)	17–88	17–76	18–88	N/A
	Mean ± SD (°)	44.0 ± 16.1	39.8 ± 12.9	63.0 ± 15.1	N/A

**Table 2:** Comparison on relative spinal dimensions of vertebrae and discs in the thoracic spine (T4-T12) in AIS subgroups and normal controls.

	Primary thoracic curves (Lenke 1–4) T4-12 (n = 86)	Secondary thoracic curves (Lenke 5–6) T4-T12 (n = 19)	Normal controls T4-T12 (n = 11)	P ANOVA between all 3 groups	P Lenke 1–4 versus Lenke 5–6	P Lenke 1–4 versus controls	P Lenke 5–6 versus controls
Vertebra *R*_*hw*_	0.67 ± 0.05	0.71 ± 0.06	0.65 ± 0.06	<**0.01**	<**0.01**	0.22	<**0.01**
Vertebra *R*_*hd*_	0.82 ± 0.06	0.85 ± 0.11	0.78 ± 0.08	0.06	0.14	0.09	<**0.05**
Vertebra *R*_*wd*_	1.23 ± 0.07	1.21 ± 0.10	1.20 ± 0.06	0.26	0.17	0.06	0.41
Disc *R*_*hw*_	0.17 ± 0.02	0.16 ± 0.02	0.15 ± 0.04	<**0.01**	<**0.05**	0.06	0.28
Disc *R*_*hd*_	0.21 ± 0.03	0.18 ± 0.02	0.17 ± 0.04	<**0.01**	<**0.01**	<**0.05**	0.26
Disc *R*_*wd*_	1.22 ± 0.07	1.19 ± 0.10	1.19 ± 0.05	0.18	0.11	0.05	0.47

Statistically significant results, *p* < 0.05 are shown in bold.

**Table 3:** Comparison on relative spinal dimensions of vertebrae and discs in the lumbar spine (L1-L5) in AIS subgroups and normal controls.

	Primary thoracic curves (Lenke 1–4) L1-L5 (n = 86)	Secondarythoracic curves (Lenke 5–6) L1-L5 (n = 19)	Normal controls L1-L5 (n = 11)	P ANOVA (between all 3 groups)	P Lenke 1–4 versus Lenke 5–6	P Lenke 1–4 versus Controls	P Lenke 5–6 versus Controls
Vertebra *R*_*hw*_	0.63 ± 0.05	0.62 ± 0.05	0.57 ± 0.09	<**0.01**	0.22	<**0.05**	<**0.05**
Vertebra *R*_*hd*_	0.89 ± 0.08	0.88 ± 0.12	0.79 ± 0.09	<**0.01**	0.48	<**0.01**	<**0.01**
Vertebra *R*_*wd*_	1.40 ± 0.08	1.42 ± 0.09	1.39 ± 0.08	0.65	0.26	0.33	0.21
Disc *R*_*hw*_	0.20 ± 0.02	0.20 ± 0.03	0.19 ± 0.03	0.95	0.47	0.42	0.45
Disc *R*_*hd*_	0.28 ± 0.02	0.28 ± 0.03	0.27 ± 0.05	0.84	0.38	0.40	0.36
Disc *R*_*wd*_	1.39 ± 0.08	1.41 ± 0.10	1.37 ± 0.09	0.49	0.20	0.32	0.17

Statistically significant results, *p* < 0.05 are shown in bold.
